# Public Perceptions of Multiple Long‐Term Conditions: An Analysis of Mass Observation Accounts

**DOI:** 10.1111/hex.70868

**Published:** 2026-09-14

**Authors:** Sue Bellass, Thomas Scharf, Victoria Bartle, Avan A. Sayer, Rachel Cooper

**Affiliations:** ^1^ AGE Research Group, Translational and Clinical Research Institute, Faculty of Medical Sciences Newcastle University Newcastle upon Tyne UK; ^2^ NIHR Newcastle Biomedical Research Centre, Newcastle upon Tyne Hospitals NHS Foundation Trust, Cumbria, Northumberland Tyne and Wear NHS Foundation Trust and Newcastle University Newcastle upon Tyne UK; ^3^ ADMISSION Research Collaborative Newcastle upon Tyne UK; ^4^ Population Health Sciences Institute, Faculty of Medical Sciences Newcastle University Newcastle upon Tyne UK

**Keywords:** Mass Observation Project, multimorbidity, multiple long‐term conditions, multiple long‐term health conditions

## Abstract

**Introduction:**

The growing number of people living with two or more long‐term conditions presents challenges to medicine and society. While the terminology of ‘multiple long‐term conditions’ (MLTC) or ‘multimorbidity’ has increasingly been adopted in research, policy and clinical practice, how the public responds to ‘MLTC’ remains underexplored. Given the important implications for research inclusion and intervention uptake, we aimed to address this gap by investigating public perceptions of MLTC.

**Methods:**

Mass Observation Project respondents were invited to share their views on MLTC. We collated the five words that respondents were asked to provide when hearing the term ‘multiple long‐term health conditions’ and compared the responses of people with and without MLTC. We then reviewed each full written account and identified categories describing the different ways in which the respondents referred to MLTC.

**Results:**

Of 141 valid responses, 76 (53.9%) were written by people living with MLTC, two thirds of whom (67.1%) were aged 60 or over. Regardless of MLTC status, respondents predominantly associated MLTC with challenging embodied experiences such as pain and debilitation. We identified four types of response to ‘MLTC’: adopting, overlooking, resisting and rejecting. Around half the respondents (51.8%) adopted the construct, with 11.3% resisting or rejecting it due to concerns about its utility, incongruence with personal experience, or medicalisation of human experiences of illness.

**Conclusion:**

MLTC were predominantly associated with challenging embodied experiences, and the construct was not adopted by all respondents. Researchers, clinicians and policymakers should carefully consider how to describe the construct of MLTC in public‐facing materials to optimise research inclusion and intervention uptake.

**Patient or Public Contribution:**

Co‐author VB was a public co‐investigator within the ADMISSION Research Collaborative. VB was involved from the outset of this project, contributing to the directive design, data interpretation workshops, critical review of the draft manuscript and approval of the final version.

Findings from the study have also been discussed with the ADMISSION Patient Advisory Group, a diverse group of patients and carers with lived experience of MLTC who provided advice to ADMISSION Research Collaborative researchers at regular intervals throughout the lifetime of the funding.

## 1 Introduction

Multiple long‐term conditions (MLTC; also referred to as multimorbidity)—the coexistence of two or more chronic health conditions—are becoming increasingly common and have consequently been identified as a key priority for research [1]. MLTC are becoming the norm among patient groups, and in recent years, a wealth of evidence has begun to accumulate to suggest the need for healthcare services to be reconfigured to better meet the needs of this growing population [2, 3, 4]. However, while the terms MLTC and multimorbidity are commonly used in clinical, research and policy spheres, they are not embedded in public consciousness with accompanying social narratives in the same way that common single conditions such as diabetes and asthma are. For example, when the Richmond Charities Taskforce on multiple conditions [5] introduced the term ‘multimorbidity’ to people living with more than one health condition, it was perceived to be a medicalised label with negative associations with bodily failure, little hope of recovery and fatality [6, 7]. In his clinical practice, Fortin (2018) similarly found that ‘multimorbidity’ failed to represent the experiences of people living with MLTC, who felt that it implied serious illness, negative judgement and physical deterioration [8]. Fortin (2018) and Stirland (2019), highlighting the importance of language use, both conclude that use of ‘multimorbidity’ should be confined to research and clinical spheres, with alternative terms such as ‘multiple chronic conditions’ promoting a more person‐centred approach in communication with patients [8, 9].

Similar terms have gained traction in the lexicon. The National Institute for Health and Care Research (NIHR), a major UK funder of health research, has expressed a preference for the term ‘multiple long‐term conditions’ [10]. This is perceived as a more optimistic descriptor, associated with longevity and ongoing management of conditions, and is now recommended for use in research and practice in the UK [7]. However, both ‘multimorbidity’ and ‘multiple long‐term conditions’ foreground the quantity of conditions, arguably masking wide‐ranging differences in health experiences [11]. While ‘multiple long‐term conditions’ may be viewed as a more palatable term and an acceptable replacement of ‘multimorbidity’, both terms may fall short conceptually when it comes to adequately reflecting MLTC lived experience [12].

While the acceptability of ‘multimorbidity’ has been subject to some scrutiny, how the public—including people living with MLTC—respond to the construct of MLTC remains underexplored. Given this has potential ramifications for intervention uptake and participation in research [13], we aimed to address this important evidence gap by analysing written accounts from members of the public who contribute to the Mass Observation Project (MOP), a British social history archive.

## 2 Materials and Methods

### 2.1 Data Collection

The MOP, originally established in the 1930s, is a social history archive that aims to capture everyday life in Britain. Three times a year, the MOP distributes a ‘directive’ (a list of questions or prompts on topics of national or global interest), inviting their panel of approximately 600 contributors to share their thoughts and opinions via written accounts. To enable comparison of different groups of respondents, the MOP ask contributors to provide biographical information including age, gender, ethnicity, location and disability status, which is anonymised and shared with research teams.

In 2023, the co‐authors commissioned a directive on MLTC. Here we report on how MOP respondents, some of whom reported living with more than one long‐term condition, perceived the term ‘multiple long‐term health conditions’. The directive was devised by the co‐authors and refined in consultation with members of the MOP team before being distributed to 597 panel members in November 2023. The directive comprised questions on experiences and perceptions of MLTC relating to awareness and understandings, everyday life, health and social care and the life course (https://massobs.org.uk/wp-content/uploads/2024/07/Autumn_-FINAL_141123-1.pdf).

In response to advice from the MOP team, the word ‘health’ was included in presenting MLTC to communicate the health focus of the directive more explicitly to the MOP respondents. We considered ‘multiple long‐term health conditions’ to be synonymous with MLTC, and a helpful means of providing clarity to the respondents, with whom we would have no communication other than via the directive. As a result, the phrase ‘multiple long‐term health conditions’ was utilised throughout the directive. We chose not to use the acronym MLTHC to avoid the risk of confusing or alienating respondents [14].

### 2.2 Defining MLTC

Recognising the challenge of introducing an unknown and inherently heterogeneous health construct to MOP respondents, we provided the Academy of Medical Sciences (2018) definition of MLTC [1] and a list of 11 long‐term health conditions as examples. These were carefully chosen to include mental, physical and long‐term infectious conditions, congenital and acquired conditions, and conditions affecting a range of body systems. Acknowledging the risk that MOP respondents may not respond if conditions they were familiar with were not listed, we emphasised that we were interested in all long‐term conditions.

### 2.3 Analysis

In this article, we present two analyses relating to public perceptions of MLTC, having first classified respondents as living with MLTC or not based on the content of their written accounts. First, we created two word clouds to summarise responses to a request to 'write down 5 words that come to mind when you hear the term “multiple long‐term health conditions”'. This allowed us to visually represent the meanings people ascribed to the term and the frequency with which those words appeared. As our aim was to summarise the words reported by the respondents, we did not use synonyms or other methods to group the responses prior to generating the word clouds.

Second, we reviewed each written account and identified categories to describe the different ways in which the MOP respondents referred to the construct in their accounts. Further analyses were then undertaken iteratively by the lead author (SB), an experienced qualitative researcher, to identify the reasons underpinning resistance to or rejection of ‘MLTC’. These analyses were discussed with the co‐authors during three data workshops to ensure a transparent, rigorous and reflexive approach.

## 3 Results

Of 146 responses received (146/597, 24.5%), 141 were considered valid and included in our analyses. Submitted typed or hand‐written accounts varied in length from a single paragraph to over 16 pages, with nearly two thirds of MOP respondents (63.1%) providing free‐form accounts rather than following the structure of the directive.

### 3.1 Characteristics of the MOP Respondents

Most respondents were women (73.8%), and over half of all respondents (54.6%) were aged 60 years or over (Table 1). Of the 109 MOP respondents who shared their ethnic identity, most identified as White or White British, one identified as British Asian and another as ‘mixed’. Twenty‐four respondents identified as a person living with a disability.

**Table 1:** Distribution of age and gender of the Mass Observation respondents (*N* = 141).

	Respondents living with MLTC (*n* = 76)		Respondents not living with MLTC (*n* = 65)		
Age (years)	Women	Men	Women	Men	Non‐binary
	*N* (%)	*N* (%)	*N* (%)	*N* (%)	*N* (%)
**20–29**	1 (1.6)	0 (0)	1 (2.3)	2 (9.5)	0 (0)
**30–39**	5 (8.2)	1 (6.7)	6 (14.0)	3 (14.3)	0 (0)
**40–49**	6 (9.8)	3 (20.0)	9 (21.0)	2 (9.5)	0 (0)
**50–59**	6 (9.8)	3 (20.0)	8 (18.6)	7 (33.3)	1 (100.0)
**60–69**	12 (19.7)	3 (20.0)	7 (16.3)	4 (19.0)	0 (0)
**70–79**	25 (41.0)	4 (26.7)	10 (23.3)	3 (14.3)	0 (0)
**80–89**	5 (8.2)	1 (6.7)	1 (2.3)	0 (0)	0 (0)
**90–99**	1 (1.6)	0 (0)	1 (2.3)	0 (0)	0 (0)
**Total**	61	15	43	21	1

#### 3.1.1 Comparison With the MOP Panel

When compared with the MOP participants who did not respond to the MLTC directive, those who did respond were, on average, older (60 vs. 55 years; *p* < 0.01). There were no marked differences in the distribution of either gender or disability status.

### 3.2 Words Associated With the Term ‘Multiple Long‐Term Health Conditions’

Of the 141 respondents, 88.2% (67/76) of the people who reported living with more than one long‐term condition and 87.7% (57/65) of the people who did not report living with MLTC, 20 of whom reported caring responsibilities for someone living with MLTC, responded to the prompt to write down five words associated with ‘multiple long‐term health conditions’.

**Figure 1:**
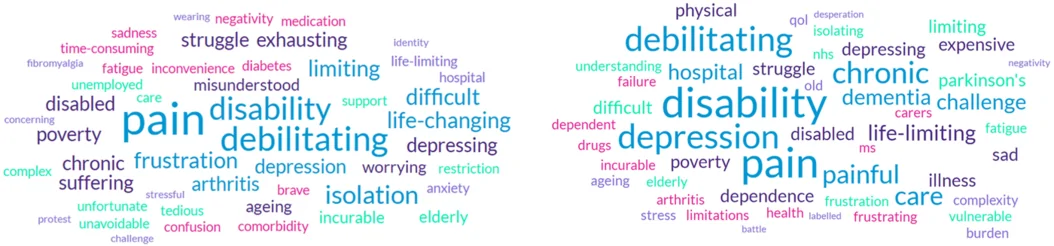
Words associated with MLTC stratified by MLTC status*. MOP respondents living with MLTC (*n* = 67). MOP respondents not living with MLTC (*n* = 57). *The size of the word is scaled to the frequency with which it was reported by different respondents.

The word clouds generated from these responses, stratified by MLTC status (Figure 1), highlight the similarity between the words selected by people living with and without MLTC. Both groups associated the term with the embodied experiences long‐term conditions might cause, primarily pain, disability and debilitation. Specific diagnoses, explicit reference to stigma and discrimination, self‐management practices, or aspects of health or social care were also mentioned albeit less often. Differences evident in the word clouds were that depression, care and burden appeared more frequently in the group without MLTC. The word cloud of people with MLTC more commonly included words highlighting challenging ways in which MLTC can affect lived lives, including ‘exhausting’, ‘life‐changing’, ‘isolation’ and ‘struggle’. Issues relating to economic matters, such as poverty, unemployment and the expense related to MLTC, were listed by people in both groups (18/124, 14.5%). Words relating to later life and ageing were infrequently mentioned, with the terms ‘ageing’ and ‘elderly’ both appearing five times, ‘old age’ twice and ‘old’ once. Virtually absent were words representing a positive response to MLTC, although ‘brave’ was included by three people and ‘positivity’, ‘resilience’, ‘overcoming’, ‘determination’, ‘acceptance’, ‘compassion’, ‘optimism’ and ‘empathy’ by single individuals.

### 3.3 Spectrum of Responses to MLTC

Categorisation of how MOP respondents chose to use the construct MLTC in their accounts demonstrated a spectrum of responses from adoption through resistance to rejection. Overall, people categorised as ‘adopting’ MLTC (73/141; 51.8%) used the phrase ‘multiple long‐term conditions’ or acronym ‘MLTC’, a synonym such as multimorbidity or multiple chronic conditions, or a variant of the acronym (such as LTMHC) at least once in their account. Of the group of people living with MLTC, 46.1% (35/76) adopted the term. For example, one respondent, a 62‐year‐old man, wrote ‘I also have a long‐term friend who definitely has a good number of multiple long‐term health conditions (hereafter referred to as “MLTHC”—to save repeatedly typing it in full!)’.

People categorised as ‘resisting’ (13/141; 9.2%) queried the relevance of the term but used it in their accounts. MOP respondents categorised as ‘rejecting’ MLTC (3/141; 2.1%) took a more unequivocal stance, challenging the validity of the term. A further group of MOP respondents (52/141, 36.9%) categorised as ‘overlooking’ MLTC made no reference to the term, which may have been an active choice or simply a preference to use more familiar terms such as a specific diagnosis or diagnoses. Within the group of people who were classified as living with MLTC, 38.2% (29/76) did not refer to the term MLTC in their accounts.

#### 3.3.1 Reasons for Resisting and Rejecting the Term ‘Multiple Long‐Term Health Conditions’

Although most MOP respondents either adopted or overlooked the term MLTC, 16 MOP respondents (11.3%), 12 of whom reported living with more than one long‐term condition, and the remaining four who witnessed MLTC in others, expressed ambivalence towards or dislike of the term. The 13 ‘resisters’ (6 women, 6 men and 1 person identifying as non‐binary), nine of whom reported living with more than one long‐term condition, were aged between 52 and 79. The three people classified as ‘rejecters’, all of whom were living with MLTC, were women aged between 39 and 78. Three patterns could be discerned in the responses: (a) challenges to the utility of the construct MLTC, (b) misalignment with identity and (c) the construct of MLTC as embedded within a broader healthcare agenda.

a. **Challenges to the utility of the construct MLTC**
  Some MOP respondents questioned the utility of the term MLTC, suggesting the construct is too abstract. One 38‐year‐old man, for instance, highlighted that the experience of MLTC would vary according to the features of the conditions and the extent to which they may limit opportunities for work, study and independent living. In his view, the term was ‘so vague as to be impossible to say anything meaningful about’. Similarly, a 58‐year‐old person identifying as non‐binary noted that the discussion would be better framed around disability due to ‘the concept being inherently abstract’. A 77‐year‐old man highlighted that a single long‐term condition could be more impactful than multiple well‐managed conditions. A clear rejection of MLTC was given by a 73‐year‐old woman who stated that the term is ‘far too amorphous to stimulate intelligent debate’.
b. **Misalignment with identity**
  While some MOP respondents offered a general challenge to the construct of MLTC, others highlighted at least three ways in which they did not perceive MLTC to be intrinsic to their identity. Typically, this was because they did not view their conditions as impinging on their everyday lives; they viewed their health as age‐related, or that they perceived MLTC to be a label that would be assigned to people who were more unwell. For example, a 76‐year‐old woman noted that her conditions were not serious in comparison with those experienced by other people. A 56‐year‐old man living with depression and anxiety would be ‘embarrassed’ to be labelled as having MLTC: ‘I would feel as though I was trying to lay claim to a status that wasn't mine’.
c. **MLTC as embedded within a broader agenda**
  Some MOP respondents expressed scepticism towards the introduction of the MLTC construct, suggesting that it may serve a broader agenda. One 52‐year‐old female writer, for example, noted that ‘anyone employed in any kind of therapeutic profession…obviously has a vested interest in telling you that you're basically falling to pieces and can't manage/survive without them’. In a similar vein, a 79‐year‐old man suggested that the purpose of placing people in a ‘scary‐sounding’ category aligns with the ‘seemingly ever‐increasing range of specialisms, each of which needs to find expanding bespoke cohorts’. Another writer, a 56‐year‐old man, queried whether the term MLTC served to medicalise human experiences of illness, pondering whether the aim was to address lack of public awareness or to improve holistic care: ‘people have always suffered from debilitating or disruptive illnesses…what is the purpose of ‘rebranding’ (if you like) a normal aspect of human experience? Is there a feeling amongst the medical community that sufferers are vulnerable to falling between the cracks of the healthcare system?’.

While the proportion of writers resisting or rejecting the term was relatively low (16/141; 11.3%), it is worth noting that 12 of the 76 people (15.8%) who reported living with more than one long‐term condition resisted or rejected ‘multiple long‐term conditions’, with potential implications for their engagement in research or interventions specifically targeting MLTC.

## 4 Discussion

Researchers and clinicians have highlighted the negative associations patients and members of the public have with the term ‘multimorbidity’ [5, 6, 7, 8, 9]. This has led to ‘multiple long‐term conditions’ becoming the recommended terminology in the UK [7]. However, to date, there has been limited focus on how members of the public perceive or engage with the term MLTC. This study aimed to investigate public perceptions of MLTC through an analysis of written accounts provided by Mass Observation participants.

The importance of understanding how people perceive and make sense of chronic illness has long been recognised [15, 16, 17, 18, 19]. Sense‐making processes occur within a nexus of social narratives and stereotypes [20], drawing on symbolic representations to shape expectations of the nature and trajectory of health conditions [21]. As a term currently largely confined to clinical, research and policy spheres, our analysis of the written accounts of MOP respondents enabled us to explore sense‐making processes when a novel health construct—MLTC—is introduced to the public.

Through the word association task, we found that MLTC was inextricably bound up with notions of pain, debilitation and limitation for MOP respondents living with MLTC, and for care and dependency for MOP respondents without MLTC. The pervasiveness of health pessimism in the responses to the task [22, 23] points to an internalisation of social norms and embodiment of stereotypes around illness [24]. With evidence suggesting that health pessimism adversely affects important life course decisions such as retirement age [25] and health outcomes [26, 27], exploring ways to create more hopeful narratives around MLTC should be prioritised. This might enable people to feel that support to manage conditions is accessible and effective. Given the well‐established association between age and MLTC in the evidence base [28, 29], pessimistic perceptions of MLTC may also compound existing and pervasive institutional, interpersonal and self‐directed ageism [30]. Reconfiguring healthcare services to respond to the challenge of MLTC and delivering healthcare services to populations with a high prevalence of MLTC, will therefore require an awareness of and response to the public perceptions of MLTC as challenging embodied experiences.

Evidence from our study suggests that MLTC (with ‘health’ added to the phrase in efforts to communicate the construct more effectively to MOP respondents), is not uncritically accepted. While just over half of MOP respondents adopted the term, over a third did not use it in their accounts. Across all the accounts, a tenth resisted or rejected the term, and this was slightly higher (15.8%) amongst respondents who reported living with more than one long‐term health condition. Respondents critiqued the construct for being too heterogeneous to be meaningful, to not adequately represent lived experience, and to have been introduced within professional spheres as part of an agenda. This extends earlier work on ‘multimorbidity’, where the critiques from people living with MLTC focussed more on associations with bodily failure, stigma and fatality than on inherent heterogeneity and perceptions of hidden agendas. Beaney (2024) argues that the introduction of the constructs of multimorbidity and MLTC represent an attempt to move away from disease‐centred models towards a person‐centred approach that recognises complexity and provides a foundation for integrated care [31]. Our study shows, however, that a term thought to be more person‐centred is not universally accepted outside of research and clinical spheres. Creating equivalent terminology that is more acceptably worded does not address the inherent conceptual tensions of heterogeneity, representativeness of lived experience, and an orientation to quantity of diagnoses.

Nonetheless, increasing MLTC prevalence and the weight of evidence demonstrating poorer outcomes associated with MLTC compared to single long‐term conditions means the construct will continue to shape healthcare delivery and health research. Our study therefore has implications for the increasing drives within health research to ensure inclusive study designs that resonate with people living with MLTC [32, 33]. Attracting potential participants to MLTC research and promoting uptake of interventions for MLTC will depend on whether people living with MLTC identify with the term. Importantly, our study suggests there may be key differences between the way MLTC is framed in professional spheres and how it is understood by people living with MLTC (or witnessing others’ lives with MLTC). People with lived experience may consider that there is an implication of severity or debilitating impact associated with the term that is not reflective of their experience or identity. Our findings therefore suggest that careful consideration needs to be given by researchers, care professionals and policymakers to communicating the construct of MLTC in clinical and research activities.

How MLTC is communicated to the public will vary according to the aims and design of the activity, but one option may be to provide a brief list of examples of the broad range of physical and mental health conditions affecting different body systems that are typically considered when defining MLTC [13]. We also advise emphasising that the list is not exhaustive and to avoid implications of severity, dependence or burden. Involving public contributors in the co‐design of patient‐ or public‐facing materials is crucial to ensure appropriate use of accessible language and optimise inclusive engagement [34].

### 4.1 Strengths and Limitations

Our study's strengths include the anonymity of the MOP approach and the trusting, sometimes decades‐long relationship between the respondents and the MOP facilitates highly candid and introspective accounts [35, 36]. In the absence of a dynamic interaction with the commissioning research team, MOP respondents may feel more at liberty to express their thoughts and opinions than in face‐to‐face research, where participants may be less likely to openly challenge the constructs utilised by researchers.

An additional strength relates to the volume and diverse nature of the data. In comparison with most qualitative research, which is generally small‐scale and geographically limited [37], we analysed over 450 pages of data from 141 MOP respondents living in 11 regions in Britain. Further, MOP respondents lived with a broad range of conditions and condition combinations, enabling us to explore MLTC as a phenomenon rather than being constrained by specific diagnostic labels. The breadth of data available offered a range of analytic avenues to pursue and we purposefully chose to focus here on summarising the responses to MLTC terminology. However, the archive presents opportunities to conduct further investigations which could privilege different aspects of the dataset.

We also acknowledge two key limitations. First, on the advice of the MOP team, we included the word ‘health’ in the construct, to explicitly communicate the nature of MLTC to MOP respondents. While respondents’ accounts may have differed had the term ‘MLTC’ been used, reflecting the accepted language in UK research [7], our view is that MOP respondents would have interpreted MLTC as pertaining to health conditions nevertheless, given the clear health focus of the directive.

A second limitation concerns the sociodemographic characteristics of the respondents whose accounts were included in this analysis. Respondents’ characteristics are not representative of the UK population as a whole; only a quarter of accounts were written by men, and over half (54.6%) by people aged 60 and over. Further, most of the respondents hold (or had retired from) professional or administrative jobs. We advise that the findings should be interpreted in light of the sample profile and suggest that future research should seek to explore the views of groups underrepresented in this study.

## 5 Conclusion

Previous work highlighting limitations in the acceptability of the term ‘multimorbidity’ to people living with more than one long‐term health condition [5] has led to increasing use of the term ‘MLTC’ in UK research, policy and practice spheres. However, findings from our study suggest that the term MLTC is neither unproblematic nor universally reflective of people's lived experience. Public perceptions of MLTC, evidenced in the word clouds, are of challenging embodied experiences. These associations may limit the extent to which people with well‐managed conditions identify with the term and, given MLTC prevalence is driven by age, further fuel institutional, interpersonal and self‐directed ageism. Understanding the spectrum of public perceptions of the construct of MLTC should facilitate a more nuanced approach to communicating MLTC in public‐ and patient‐facing materials to optimise research inclusion and intervention uptake.

## Author Contributions

**Sue Bellass:** conceptualisation, writing – original draft, writing – review and editing, investigation, data curation, formal analysis, project administration. **Thomas Scharf:** funding acquisition, conceptualisation, writing – review and editing, investigation, formal analysis, supervision. **Victoria Bartle:** conceptualisation, writing – review and editing, investigation, formal analysis. **Avan A. Sayer:** funding acquisition, conceptualisation, writing – review and editing, investigation, formal analysis. **Rachel Cooper:** funding acquisition, conceptualisation, writing – review and editing, investigation, formal analysis, supervision.

Sue Bellass, Avan A. Sayer and Rachel Cooper also acknowledge support from the National Institute for Health and Care Research (NIHR) Newcastle Biomedical Research Centre (grant reference NIHR203309).

The funders played no role in the design of the study and collection, analysis, and interpretation of data or in writing the manuscript. The views expressed in this publication are those of the authors and not necessarily those of UK Research and Innovation, the National Institute for Health and Care Research or the Department of Health and Social Care.

## Ethics Statement

All co‐authors were granted permission by the Mass Observation Project management team, based at the University of Sussex, to access the anonymised data described for secondary analyses.

## Patient Consent Statement

All Mass Observation Project respondents sign a copyright form that provides consent for their writing to be accessed by the public, published and used in any social media posts or communications. Respondents are provided with a privacy policy which describes how their data are stored and managed. Only anonymised data are made available to external research teams. All accounts returned to Mass Observation are checked for identifiable material and redacted where necessary.

## Conflicts of Interest

The authors declare no conflicts of interest.

## Permission to Reproduce Material From Other Sources

Mass Observation material reproduced with permission of Curtis Brown Group Ltd, London, on behalf of the Trustees of the Mass Observation Archive. Copyright (c) The Trustees of the Mass Observation Archive.

## Data Availability

The data that support the findings of this study are available from Mass Observation Project. Restrictions apply to the availability of these data, which were used under license for this study. Data are available from https://massobs.org.uk/ with the permission of Mass Observation Project. The data analysed for this article are archived and available through application to the Mass Observation Archive moa@sussex. ac. uk.

## References

[1] Academy of Medical Sciences , *Multiple Long‐Term Conditions (Multimorbidity): A Priority for Global Health Research* (Academy of Medical Sciences, 2018).

[2] K. Barnett , S. W. Mercer , M. Norbury , G. Watt , S. Wyke , and B. Guthrie , “Epidemiology of Multimorbidity and Implications for Health Care, Research, and Medical Education: A Cross‐Sectional Study,” Lancet 380, no. 9836 (2012): 37–43, 10.1016/S0140-6736(12)60240-2. 22579043

[3] B. Guthrie , K. Payne , P. Alderson , M. E. T. McMurdo , and S. W. Mercer , “Adapting Clinical Guidelines to Take Account of Multimorbidity,” BMJ (London) 345 (2012): e6341, 10.1136/bmj.e6341. 23036829

[4] C. Salisbury , M. S. Man , P. Bower , et al., “Management of Multimorbidity Using a Patient‐Centred Care Model: A Pragmatic Cluster‐Randomised Trial of the 3D Approach,” Lancet 392, no. 10141 (2018): 41–50, 10.1016/s0140-6736(18)31308-4. 29961638 PMC6041724

[5] Richmond Group of Charities ,*You Only Had to Ask: What People With Multiple Conditions Say About Health Equity* (Richmond Group of Charities, 2021).

[6] C. Chew‐Graham , L. O'Toole , J. Taylor , and C. Salisbury , “‘Multimorbidity’: An Acceptable Term for Patients or Time for a Rebrand?,” British Journal of General Practice 69, no. 685 (2019): 372–373, 10.3399/bjgp19X704681. PMC6650114 31345795

[7] K. Khunti , H. Sathanapally , and P. Mountain , “Multiple Long Term Conditions, Multimorbidity, and Co‐Morbidities: We Should Reconsider the Terminology We Use,” BMJ (London) 383 (2023): p2327, 10.1136/bmj.p2327. 37832952

[8] M. Fortin , “It‐That‐Must‐Not‐Be‐Named,” Canadian Family Physician 64, no. 12 (2018): 881–882. 30541799 PMC6371864

[9] L. E. Stirland , “‘Multimorbidity’: An Acceptable Term for Patients or Time for a Rebrand?,” British Journal of General Practice (2019), https://bjgp.org/content/%E2%80%98multimorbidity%E2%80%99-acceptable-term-patients-or-time-rebrand. 10.3399/bjgp19X704681 PMC6650114 31345795

[10] "National Institute for Health and Care Research Strategic Framework for Multiple Long‐Term Conditions," Version 2.0 Sep 2024, NIHR, accessed July 11, 2026.

[11] S. Pati , C. Macrae , D. Henderson , D. Weller , B. Guthrie , and S. Mercer , “Defining and Measuring Complex Multimorbidity: A Critical Analysis,” British Journal of General Practice 73, no. 733 (2023): 373–376, 10.3399/bjgp23X734661. PMC10405940 37500453

[12] T. Porter , B. N. Ong , and T. Sanders , “Living With Multimorbidity? The Lived Experience of Multiple Chronic Conditions in Later Life,” Health: An Interdisciplinary Journal for the Social Study of Health, Illness and Medicine 24, no. 6 (2020): 701–718, 10.1177/1363459319834997. 30895825

[13] S. Bellass , A. Ellwood , S. Arakelyan , et al., “Recommendations for Conducting Qualitative Research on Multiple Long‐Term Conditions (MLTC) From a Qualitative Methods Community of Practice,” Journal of Multimorbidity and Comorbidity (2026): 16, 10.1177/26335565261459508. PMC13254441 42291122

[14] S. Zarshenas , J. Mosel , A. Chui , et al., “Recommended Characteristics and Processes for Writing Lay Summaries of Healthcare Evidence: A Co‐Created Scoping Review and Consultation Exercise,” Research Involvement and Engagement 9, no. 1 (2023): 121, 10.1186/s40900-023-00531-5. 38124104 PMC10734197

[15] M. Bury , “Chronic Illness as Biographical Disruption,” Sociology of Health & Illness 4, no. 2 (1982): 167–182, 10.1111/1467-9566.ep11339939. 10260456

[16] K. Charmaz , “Struggling for a Self: Identity Levels of the Chronically Ill,” in Research in the Sociology of Health Care, eds. J. P. Roth and P. Conrad (JAI Press, 1987).

[17] A. W. Frank , The Wounded Storyteller: Body, Illness and Ethics (Chicago University Press, 1995).

[18] M. Franklin , K. Willis , S. Lewis , and L. Smith , “Chronic Condition Self‐Management Is a Social Practice,” Journal of Sociology 59, no. 1 (2023): 215–231, 10.1177/14407833211038059.

[19] J. N. Gunning and J. Koenig Kellas , ““Being Sick Does Not Define You”: Communicated Narrative Sense‐Making of Autoimmune Disease in Emerging Adult Women in the US,” Emerging Adulthood 12, no. 2 (2024): 175–186, 10.1177/21676968231217781.

[20] R. Savolainen , “The Sense‐Making Theory: Reviewing the Interests of a User‐Centered Approach to Information Seeking and Use,” Information Processing & Management 29, no. 1 (1993): 13–25, 10.1016/0306-4573(93)90020-E.

[21] T. Sanders , K. Fryer , M. Greco , C. Mooney , V. Deary , and C. Burton , “Explanation for Symptoms and Biographical Repair in a Clinic for Persistent Physical Symptoms,” SSM. Qualitative Research in Health 5 (2024): 100438, 10.1016/j.ssmqr.2024.100438. 38915733 PMC11195018

[22] C. van Doorn , “A Qualitative Approach to Studying Health Optimism, Realism, and Pessimism,” Research on Aging 21, no. 3 (1999): 440–457, 10.1177/0164027599213005.

[23] P. Brown and S. Vickerstaff , “Health Subjectivities and Labor Market Participation: Pessimism and Older Workers’ Attitudes and Narratives Around Retirement in the United Kingdom,” Research on Aging 33, no. 5 (2011): 529–550, 10.1177/0164027511410249.

[24] A. Zajacova , H. Grol‐Prokopczyk , and Z. Zimmer , “Sociology of Chronic Pain,” Journal of Health and Social Behavior 62, no. 3 (2021): 302–317, 10.1177/00221465211025962. 34283649 PMC8956223

[25] M. van der Horst , “Internalised Ageism and Self‐Exclusion: Does Feeling Old and Health Pessimism Make Individuals Want to Retire Early?,” Social Inclusion 7, no. 3 (2019): 27–43, 10.17645/si.v7i3.1865.

[26] H. J. Craig , J. Ryan , R. Freak‐Poli , et al., “Dispositional Optimism and All‐Cause Mortality in Older Adults: A Cohort Study,” Psychosomatic Medicine 83, no. 8 (2021): 938–945, 10.1097/psy.0000000000000989. 34334727 PMC8490272

[27] M. F. Scheier , J. D. Swanson , M. A. Barlow , J. B. Greenhouse , C. Wrosch , and H. A. Tindle , “Optimism Versus Pessimism as Predictors of Physical Health: A Comprehensive Reanalysis of Dispositional Optimism Research,” American Psychologist 76, no. 3 (2021): 529–548, 10.1037/amp0000666. 32969677

[28] J. Valabhji , E. Barron , A. Pratt , et al., “Prevalence of Multiple Long‐Term Conditions (Multimorbidity) in England: A Whole Population Study of Over 60 Million People,” Journal of the Royal Society of Medicine 117, no. 3 (2024): 104–117, 10.1177/01410768231206033. 37905525 PMC11046366

[29] A. Head , K. Fleming , C. Kypridemos , J. Pearson‐Stuttard , and M. O'Flaherty , “Multimorbidity: The Case for Prevention,” Journal of Epidemiology and Community Health 75 (2021): 242–244. 33020144 10.1136/jech-2020-214301 PMC7892394

[30] World Health Organisation , Global Report on Ageism (World Health Organisation, 2021).

[31] T. Beaney , “Is Consensus Attainable on the Definition of Multiple Long Term Conditions?,” BMJ (London) 384 (2024): q230, 10.1136/bmj.q230. 38453185

[32] S. Staniszewska , S. Denegri , R. Matthews , and V. Minogue , “Reviewing Progress in Public Involvement in NIHR Research: Developing and Implementing a New Vision for the Future,” BMJ Open 8, no. 7 (2018): e017124, 10.1136/bmjopen-2017-017124. PMC6067369 30061427

[33] A. M. Biggane , M. Olsen , and P. R. Williamson , “PPI in Research: A Reflection From Early Stage Researchers,” Research Involvement and Engagement 5, no. 1 (2019): 35, 10.1186/s40900-019-0170-2. 31832239 PMC6865031

[34] National Institute for Health and Care Research . Research Inclusion Strategy 2022‐2027 (National Institute for Health and Care Research, 2022), https://www.nihr.ac.uk/about-us/who-we-are/research-inclusion/strategy-2022-27.

[35] V. May , “When Recognition Fails: Mass Observation Project Accounts of Not Belonging,” Sociology 50, no. 4 (2016): 748–763.

[36] J. Scantlebury and K. Pattrick , “Mass Observing COVID‐19,” in Ethics of Contemporary Collecting, eds. J. Kavanagh , E. Miles , R. L. West , and S. Cordner (Routledge, 2024), 1st ed., 75–86.

[37] A. Pollen , “Research Methodology in Mass Observation Past and Present: ‘Scientifically, About as Valuable as a Chimpanzee's Tea Party at the Zoo’?,” History Workshop Journal 75, no. 75 (2013): 213–235.

